# Design and Calibration of a Low-Cost SDI-12 Soil Moisture Sensor

**DOI:** 10.3390/s19030491

**Published:** 2019-01-25

**Authors:** Juan D. González-Teruel, Roque Torres-Sánchez, Pedro J. Blaya-Ros, Ana B. Toledo-Moreo, Manuel Jiménez-Buendía, Fulgencio Soto-Valles

**Affiliations:** DSIE, Technical University of Cartagena, Campus Muralla del Mar s/n, Cartagena E-30202, Spain; roque.torres@upct.es (R.T.-S.); pedro.blaya@upct.es (P.J.B.-R.); ana.toledo@upct.es (A.B.T.-M.); manuel.jimenez@upct.es (M.J.-B.); pencho.soto@upct.es (F.S.-V.)

**Keywords:** soil moisture, Precision Agriculture, sensor calibration, SDI-12, volumetric water content, capacitive sensor, dielectric measurement, Smart Agriculture

## Abstract

Water is the main limiting factor in agricultural production as well as a scarce resource that needs to be optimized. The measurement of soil water with sensors is an efficient way for optimal irrigation management. However, commercial sensors are still too expensive for most farmers. This paper presents the design, development and calibration of a new capacitive low-cost soil moisture sensor that incorporates SDI-12 communication, allowing one to select the calibration equation for different soils. The sensor was calibrated in three different soils and its variability and accuracy were evaluated. Lower but cost-compensated accuracy was observed in comparing it with commercial sensors. Field tests have demonstrated the temperature influence on the sensor and its capability to efficiently detect irrigation and rainfall events.

## 1. Introduction

Several variables have a great influence on crop development. Control and knowledge of them allows a better management of necessary resources, as the term ‘Precision Agriculture’ points out [1,2,3]. This knowledge enhances the economic and ecologic performance, while providing a better comprehension of crop behavior. The soil is a fundamental element in the development of any crop. Its properties to retain and drain water as well as the supply of nutrients are essential. For this reason, the understanding of the dynamics of water in the soil from variables such as Volumetric Water Content (VWC) is very useful to estimate its water status and manage irrigation efficiently [4]. 

Multiple techniques and devices have been used to determine the soil water content. These techniques can be divided in classical and modern [5,6]. Classical techniques include the feel method, which consists of estimating the soil moisture by squeezing the soil in the palm of a hand [7]; the calcium carbide technique, which measures the gas pressure generated due to chemical reaction of calcium carbide reagent with the water present in the soil [6] and the thermo-gravimetric method, which is known as the reference method. It consists of drying a wet soil sample in a convection oven at 110 ± 5 °C for 24 h and measuring the weight of the sample before and after drying [8,9].

Modern techniques include a variety of methods. One of these methods is neutron scattering, that has been used by many researchers [10,11,12]. Its principle is based on the proportion of neutrons that hit hydrogen atoms. Another technique is the Wenner method, which consists of measuring the soil resistivity using four electrodes [13,14]. The dependence between resistivity and water content of the soil is leveraged in this case. However, it is well known that this method is highly dependent on soil salinity [14,15]. Tensiometers are other widely used devices for soil moisture measurement. They consist of a water column equipped with a ceramic cup at the bottom, so that as the soil dries suction is generated on the water column [16,17]. The suction rate is related with the soil water potential [18]. Besides, thermal and optical techniques are based on the relationship between the soil water content and the thermal conductivity [19,20,21] or the reflection and refraction of the light in the soil [22,23,24], respectively.

Many methods have been developed to measure soil water content. However, among these methods, dielectric techniques stand out for their automation capability and on-site measurements, in addition to their high accuracy, their wide range of VWC and its easy installation [25,26,27]. The principle of dielectric meters is based on the great difference between the dielectric constants of air (ε_r_ ≈ 1) and water (ε_r_ ≈ 80) [5,7,28,29,30,31]. The presence of water in the soil generates a variation on its permittivity, also modifying the capacity of the soil-sensor capacitor. The disadvantage of using dielectric techniques for determining the soil water content is that VWC and soil permittivity relationship have a heavy dependence on soil texture [32]. This means that a specific calibration procedure is needed for each soil texture [26,33,34]. 

Dielectric techniques are divided into time-domain and frequency-domain techniques. Time Domain Reflectometry (TDR) determines the apparent permittivity of the soil by measuring the travel time for a pulsed electromagnetic signal along a parallel waveguide [5,6,28,35,36,37]. In the case of frequency domain techniques, two main principles can be found: Frequency Domain Reflectometry (FDR) and capacitance [6,7,38]. The former encompasses a series of methodologies that use a voltage standing wave in frequency domain, as a result of an incident and reflected signal. Depending on the distinguishing parameter selected, some establish relationship between permittivity and resonance frequency [25,38,39], whereas other relate the permittivity with the reflection and transmission coefficient [40,41,42,43,44,45,46,47,48,49]. Regarding capacitance techniques, the permittivity of the medium is determined by measuring the charge time of a capacitor which modifies the operating frequency of an oscillator. This capacitor consists of two electrodes, keeping the soil as the dielectric between them. 

The use of high frequencies by TDR technique involves more accurate VWC measurements, since the influence of salinity is reduced. However, high frequencies imply more expensive equipment [50]. Besides, using frequency domain techniques leads to an equipment cost reduction at the expense of a lower, but admissible decrease of the measurement accuracy with respect to TDR [6]. Nevertheless, commercial frequency domain sensors remain costly for medium and small farmers, creating an economic barrier and limiting the establishment of Precision Agriculture. Moreover, unlike other soil moisture measurement techniques like neutron scattering probe, that covers large measurement volumes, dielectric sensors have a reduced measurement volume of influence. Consequently, the heterogeneous pore size of the soil can lead to non-representative VWC measurements. Hence, using numerous distributed sensors in a network that offer an average measurement and integrate a representative volume is necessary to achieve an accurate result with dielectric sensors [29,33]. A cost reduction of these sensors is indispensable for reaching this goal.

Several authors have developed low-cost frequency domain sensors [26,51,52,53,54,55]. Nonetheless, the necessity of soil specific calibration is still a constraining factor. Commercial sensors like the Hydra Probe II (Stevens Water Monitoring Systems Inc., Portland, OR, USA) and the EnviroSCAN probe (Sentek Pty. Ltd., Stepney, SA, Australia) are able to select and program calibrations for different soils to obtain a higher level of accuracy [34], however, its cost limits its use to research applications. Therefore, the main objective of the work presented in this paper is the development of a low-cost soil moisture sensor that can be widely used in Precision Agriculture. In order to achieve this, the most important challenges to be addressed are how to obtain a low-cost sensor and to make it as flexible as possible in order to make the work easier for the farmer. In this sense, it is important that the sensor is operative in different types of soil and can be adapted to the different conditions and measuring equipment used. Hence, this paper presents the design and calibration of a new low-cost capacitive soil moisture sensor with flexible connection to dataloggers via an analog output and an SDI-12 communication protocol [56],which is widely used in Precision Agriculture and supported by the most popular commercial dataloggers. Unlike other low-cost developments, the sensor presented allows a flexible record and selection of calibration equations for each soil texture previously registered, in addition to the net VWC measurement. The developed device has been calibrated in three different soils and the corresponding calibration curves have been obtained. Additionally, repeatability and reproducibility of the sensor have been evaluated. The specific electronic design of the sensor and the SDI-12 programming code are described next, as well as the soil specific calibration protocol implemented.

## 2. Materials and Methods

### 2.1. Electronic Design of the Sensor

**Figure 1:**
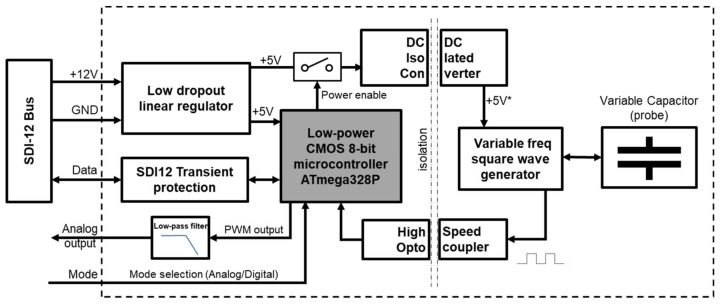
Block diagram.

Different prototypes of the sensor were developed at the Technical University of Cartagena, in Spain. Figure 1 depicts the internal functional blocks of the latest version of the moisture sensor, which integrates the measurement probe and all the electronic devices required for its operation.

The system developed is based on the picoPower 8-bit AVR RISC-based microcontroller from Microchip (Chandler, AZ, USA) which combines 32 kB ISP flash memory with read-while-write capabilities, 1 kB EEPROM, 2 kB SRAM and 23 general purpose I/O lines. The microcontroller is responsible for managing low power modes, activating the oscillator power supply, measuring the frequency of the generated signal and providing calibrated VWC measurements.

**Figure 2:**
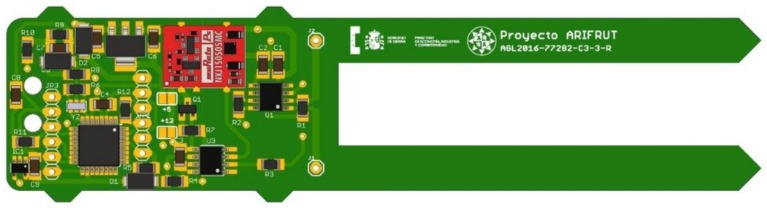
Experimental sensor PCB.

The sensing principle of the sensor is based on the soil dielectric constant variation with the VWC. As in many other research works [29,51,57,58], an oscillator has been used to determine capacitive changes in the soil. In this case, an oscillator consisting of an integrated circuit 555 in an astable configuration has been selected for economic purposes, so that this circuit generates a square wave whose frequency varies with the capacity of the probe. The probe is integrated in the sensor PCB and consists of two copper tracks along two prongs, as it is shown in Figure 2. This configuration is common in commercial capacitive soil moisture sensors [59,60,61]. The PCB was designed using four layers to guarantee the isolation from the soil. This probe geometry was tested and characterized in previous works of our research group [62], where the prongs dimensions were defined to assure a balance between integrated measurement volume and stiffness. The sensor schematic is presented in Appendix A.

The early tests showed that alterations occurred in the sensor response when installed close to other equipment or when the soil sample was grounded. Thus, all measurement electronics of the probe were electrically isolated from the rest of the components to avoid earth leakage currents that may affect the measurement in the presence of other sensors and nearby measurement equipment [63]. For this purpose, an isolated DC/DC converter was used to power the oscillator, and a high-speed optocoupler for its output signal.

The sensor can be powered from a voltage source in the range of 5.5 to 15 V. In this way it can be integrated into SDI-12 networks operating at 12 V. A low-dropout low-quiescent current voltage regulator was used to supply the 5 V required for the microcontroller and the isolated converter that feeds the measuring section. To reduce consumption, the microcontroller remains in deep sleep mode and only wakes up when the measurement process starts, activating the power supply of the oscillator.

To allow its use in a wider range of applications, the sensor can operate in two modes: as a standalone sensor with analog output or in digital mode using SDI-12 protocol. The operating mode is selected via an external pin. In analog mode the sensor provides a continuous 0 to 5 V output proportional to the VWC (0–100%). A Pulse-Width Modulation (PWM) output of the microcontroller at a frequency of 62 kHz was used for this purpose, which was filtered with a passive low-pass filter that is connected to a rail-to-rail operational amplifier in voltage follower mode to achieve a low output impedance. In digital mode, measurements are provided in response to SDI-12 commands that will be described in the next section. The sensor includes a transient protection against electrical disturbances on the bus line. In addition, some sensor connector pins have dual functionality to allow firmware reprogramming and code debugging.

### 2.2. Software Design of the Sensor

The device software was developed in C language with the Arduino integrated development environment (IDE) [64]. This environment is a cross-platform application written in Java and based on Processing and other open-source software.

**Figure 3:**
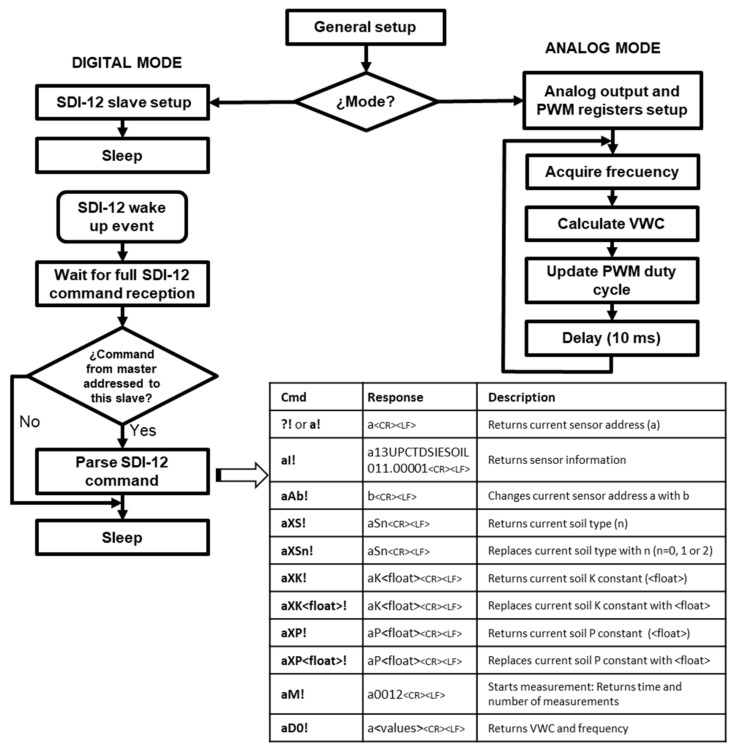
Sensor software flowchart.

Figure 3 shows the general flowchart of the sensor microcontroller program. Firstly, a general configuration is performed to read the soil type from the EEPROM memory and set up the digital I/O pins. Secondly, the mode selection pin is checked to enter the corresponding working mode.

In digital mode, the sensor operates as a slave in an SDI-12 network. The library [65] has been used for this purpose. This library provides functions to implement version 1.3 of the SDI-12 communication protocol without the need for additional hardware. In this mode, the sensor program initializes the SDI-12 slave and switches to sleep mode waiting for commands to be received via the digital bus. When an SDI12 wake up event occurs (every SDI-12 command starts with an event of this type), it triggers an interrupt that wakes up the sensor from sleep mode. The sensor waits then to receive the full command. If the command is from a master and the destination address matches the sensor address, the command is processed as shown in Figure 3.

The measurement process starts when command aM! is received. The sensor responds sending the time for the measurements to be available (1 s) and the number of measurements (in this case two: VWC and frequency). Five values of the frequency generated by the square wave generator are acquired, the maximum and minimum are suppressed and the remaining ones are averaged. The [66] library has been used to configure the internal timers and counters of the microcontroller to allow frequency measurement of square waves up to 8 MHz. The VWC is obtained from the average frequency using the calibration equation and applying the specific parameters corresponding to the selected soil.

The SDI-12 master waits for the time indicated by the sensor and sends an aD0! command to request the measurement. The sensor responds sending the VWC and frequency values. Extended commands have been implemented for the query and selection of the soil type (command aXS!) as well as for the addition of new coefficients to characterize soils other than preset ones (commands aXP! and aXK!). In addition, the sensor implements the response to other commands of the SDI-12 protocol, such as changing the slave’s address or requesting information (see Figure 3).

In analog mode, a pin is set as a PWM digital output configured at a frequency of 62.5 kHz. Next, a loop including frequency measurement, VWC calculation and generation of the PWM output corresponding to the VWC value is started. For the acquisition of the average frequency and the calculation of VWC, the same functions as in the digital mode are used. Changing the soil type or the calibration equation coefficients can only be done by switching to digital mode using the commands described above.

### 2.3. Experimental Settings

#### 2.3.1. Laboratory Experiments

The sensor has been experimentally tested and calibrated. For this purpose, the electronics were isolated and protected via an epoxy resin encapsulation. A specific mould for the encapsulation was designed in SolidWorks (Dassault Systèmes SolidWorks Corporation, Waltham, MA, USA) and built with a CR-10S Fused Deposition Modeling (FDM) 3D printer (Shenzhen Creality 3D Technology Co., Ltd., JinChengYuan, Tongsheng Community, Dalang, Longhua District, Shenzhen, China).

As described before, dielectric sensors need a soil-specific calibration due to the different porosity of the textures. Many commercial sensors are provided with a general calibration equation which relates the VWC either with the apparent permittivity (ε_a_) or the output magnitude unit of the sensor. However, for an accuracy enhancement it is usually recommended performing a soil specific calibration. For example, Decagon Devices, Inc. USA (METER Group, Inc., Pullman, WA, USA) soil moisture devices are normally said to be ±1–2%VWC accurate with a specific calibration instead of ±3%VWC with the general equation [67,68]. 

In this case, since the output magnitude of the designed sensor is the frequency, a relationship between this variable and VWC has to be established. In this way, the ECH_2_O (Decagon Devices, Inc.) soil moisture sensors calibration procedure [9] has been followed. This protocol is in turn based on the Starr and Paltineanu one [69] and it has been chosen due to the similarities between the ECH_2_O sensors and the one presented here. The procedure consists of measuring the VWC of several soil samples by means of the reference method, i.e., the thermo-gravimetric method, and relating them with the electric measurements obtained with the sensor. The samples range from dry to saturation states of the soil. Therefore, some calibration points are obtained allowing the generation of a calibration curve. Polynomial, exponential, Fourier approximation, rational and potential expressions have been evaluated to obtain the best fitted calibration curve. The selection criterion was the highest determination coefficient (R^2^) together with the fidelity between the real moisture-frequency relation and that obtained with the fit. The sensor was calibrated in three different soils: (i) clay-loam soil from area 1, (ii) clay-loam soil from area 2 and (iii) sandy soil, being area 2 more clayey than area 1. In relation with the Decagon Devices calibration procedure, the equipment used have been a cylindrical calibration container for each soil (0.275 m Ø, 0.3 m height), a PVC cylindrical volumetric soil sampler (0.036 m Ø, 0.06 m height), several aluminium soil drying containers, a mass balance (0.0001 kg resolution, 0–3 kg range) and a convection drying oven. As data acquisition system, a CR1000 datalogger (Campbell Scientific Ltd., Logan, UT, USA) together with Loggernet software was employed.

During calibration, two experimental sensors were used in order to determine their repeatability and reproducibility. Three repeated measurements at least were performed in every soil sample with both sensors. Each repetition implies extracting the sensor from the soil and reintroduce it in other undisturbed soil point. This means that the measurand changes due to the heterogeneity of the soil. Therefore, this aspect must be taken into account as a source of variability among measurements and it is unavoidable. The Repeatability and Reproducibility (R&R) study was conducted by a one factor Analysis of Variance (ANOVA) test [70,71,72]. The one factor considered is the use of two units of the experimental sensor (Arifrut1 and Arifrut2). The ANOVA test was applied for each soil sample at every moisture level tested. Moreover, variability estimators associated with repeatability and reproducibility have been defined [73]:

(1) $$\sigma_{\mathrm{repeatability}}\;=\;\sqrt{{\mathrm{MS}}_{\mathrm{ii}}},$$

(2) $$\sigma_{\mathrm{reproducibility}}\;=\;\sqrt{\frac{\left|{\mathrm{MS}}_{\mathrm{ij}}{-\mathrm{MS}}_{\mathrm{ii}}\right|}{n}},$$

(3) $$\sigma_{\mathrm{sensor}}\;=\;\sqrt{\sigma_{\mathrm{repeatability}}^{2}{+\sigma }_{\mathrm{reproducibility}}^{2}},$$

(4) $$\%\;\mathrm{Repeatability}\;=\;100\frac{\sigma_{\mathrm{repeatability}}^{2}}{\sigma_{\mathrm{sensor}}^{2}},$$

(5) $$\%\;\mathrm{Reproducibility}\;=\;100\frac{\sigma_{\mathrm{reproducibility}}^{2}}{\sigma_{\mathrm{sensor}}^{2}},$$

where $\sigma_{\mathrm{repeatability}}$ and $\sigma_{\mathrm{reproducibility}}$ are the variability estimators corresponding to repeatability and reproducibility respectively, $\sigma_{\mathrm{sensor}}$ is the general variability associated to the sensor, ${\mathrm{MS}}_{\mathrm{ii}}$ is the mean square error among the measurements of the same sensor, ${\mathrm{MS}}_{\mathrm{ij}}$ is the mean square error among the measurements of the two sensors, n is the number of observations and % Repeatability and % Reproducibility are the corresponding percentages of the whole variation associated with repeatability and reproducibility, respectively. 

#### 2.3.2. Experimental Plot Evaluation

The experimental sensor was tested under field conditions to check its capability for detecting irrigation episodes or the evolution of water across the soil. The experiments were conducted during one month. The site is located at the Agricultural Science Center Tomás Ferro (37°35’ N, 0°59’ W), property of the Technical University of Cartagena, in the south east of Spain. A deep (>2 m) and homogeneous silt–clay-loam soil (ii) with low organic matter content (1.54%) and high phosphorous content (124.2 mg kg^−1^) characterizes the installation. The soil has a water-holding capacity of about 0.18 m^3^ m^−3^ and bulk density varying within the range 1.30–1.55 g cm^−3^.

The experimental sensor was installed in a 3-year-old orchard of sweet cherry (*Prunus avium L. cv. Lapins*) grafted on Mirabolano roostock. The installation was performed at 15 cm from different representative drippers and 25 cm deep, under the canopy projection. One MPS-6 (Decagon Devices, Inc.) sensor was also installed in the same conditions paired with the experimental one. A single lateral per tree row with three 2.2 l h^−1^ auto-compensating drippers per tree is used. The irrigation was based on the water needs of the crop. 

All the data were registered by a CR1000 datalogger every 10 minutes and remotely monitored via a wireless Virtual Private Network (VPN) for permanent access to information. Rainfall data were obtained every hour from the Agricultural Information System of Murcia (SIAM-IMIDA) portal [74]. Matlab (The MathWorks, Inc., Natick, MA, USA) was used for data processing, obtaining the calibration equations and to perform the R&R study. Result plots were drawn by Matlab and Sigmaplot (Systat Software Inc., San Jose, CA, USA).

## 3. Results and Discussion

### 3.1. Sensor Variability Study

**Figure 4:**
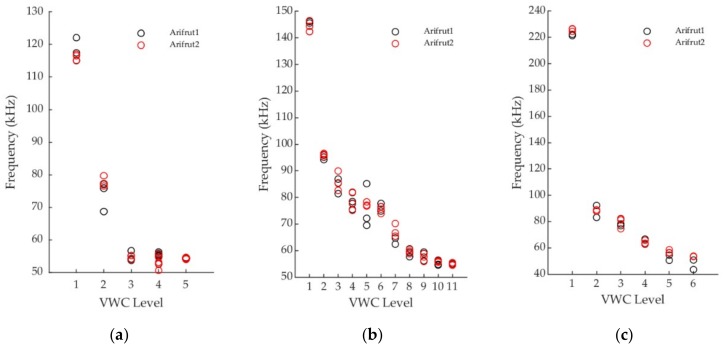
Sensor variability at different VWC levels tested in: (**a**) soil (i), (**b**) soil (ii) and (**c**) soil (iii).

In Figure 4 the different measurements obtained with the two experimental sensors (labelled Arifrut1 and Arifrut2) in laboratory tests are compared for every soil. All the VWC levels tested in calibration are presented. More calibration points of the soil (ii) were registered in order to have a more detailed behavior of the sensor in the soil to be studied during field tests. In general, the variability in the Arifrut1 sensor is more pronounced if a comparison among measurements at the same VWC level is made, although it depends on the kind of soil and the VWC level. Arifrut2 variability is negligible in the vast majority of VWC levels. Regarding the sensor-to-sensor variability, the differences are only substantial when Arifrut1 variability is marked. However, if a means comparison is made, similar response of both sensors is accomplished. The one factor ANOVA test results confirm that there are not significant differences between Arifrut1 and Arifrut2 mean values. Table 1 shows the *p*-value obtained for each ANOVA test. Since no *p*-value less than 0.01 was obtained, the similarity between the two sensors can be assumed for a significance level of α = 0.1.

The variability estimators of the experimental sensor are also presented in Table 1. The general variability of the sensor reaches its minimum values for clay-loam soils (soils (i) and (ii)) at the highest VWC levels. This performance is probably due to the fact that the wet soil behaves more homogeneously and the cohesion among its particles allows better soil-sensor contact. In the case of soil (iii) no specific relation between VWC level and variability is observed. The maximum variability is ranged from 3 to 4 kHz for every soil, considering the soil (ii), VWC level 5 as an outlier. The influence of repeatability and reproducibility on the whole variability is unbalanced, since repeatability usually accounts for at least 75% of variability. Variability therefore has lack of repeatability as its main cause. Comparable results for all the soils are obtained. 

### 3.2. Sensor Calibration

**Figure 5:**
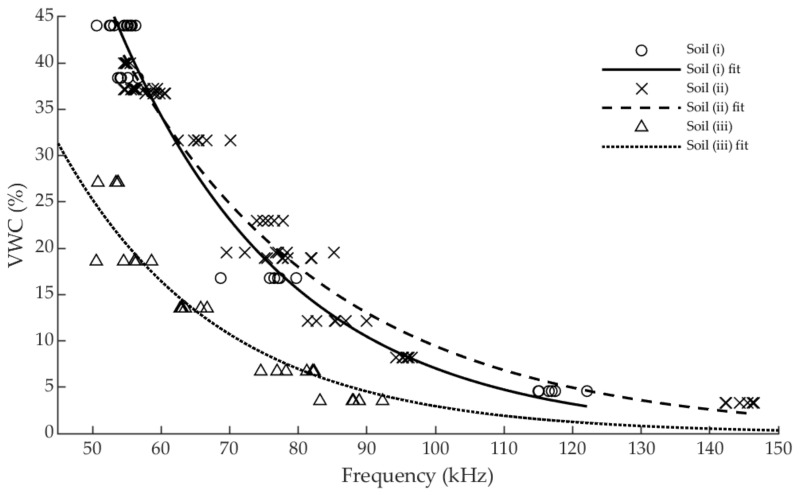
Sensor measurements and calibration curves in the three soils.

Since no significant differences between sensors Arifrut1 and Arifrut2 were determined by the ANOVA test, both sensors data were combined in order to acquire a standard calibration for each soil. Several expressions were tested to obtain the calibration curve and finally the exponential one was found to be the best option to reproduce the sensor evolution in the three soils, as it is shown in Figure 5. Second degree and higher polynomial and Fourier approximation ones are not suitable expressions in this case, since they are no injective functions along the sensor working range. Thus, only exponential, potential and rational expressions have been compared. Table 2 shows the fitting goodness results and the parameters of the calibration functions. The lowest RMSE and highest R^2^ values are achieved with the exponential function in every soil, so that it was selected as the calibration curve for the three soils.

In Figure 5 the sensor measurements and the corresponding exponential fitting curves are presented. A distinguished difference in sensor response between sandy and loamy-clay soils is noticed, demonstrating the influence of soil texture on capacitive sensors behavior. Besides, the data collected in the two clay loam soils are quite similar, although different calibration parameters are obtained. In addition, it is observed that the soil (i) saturates at a higher moisture level. While soil (ii) reaches the saturation level at practically VWC = 40%, soil (i) does so at approximately 45%. In the case of soil (iii), saturation is reached much earlier, i.e., between 25 and 30%. The exponential calibration curve provides the sensor a higher sensibility as the VWC is increased. This behavior is advantageous as the sensor will usually work above field capacity and sensitivity is only reduced below 5%VWC. However, the sensor ceases to be operational when the volumetric content exceeds the soil saturation level, as it has not been calibrated above this point. In addition, the proximity to the full scale of the sensor when the soil saturation states are reached indicates that its use is not suitable for measuring in media with a dielectric constant close to that of water, as is the case with hydroponic crops. 

**Figure 6:**
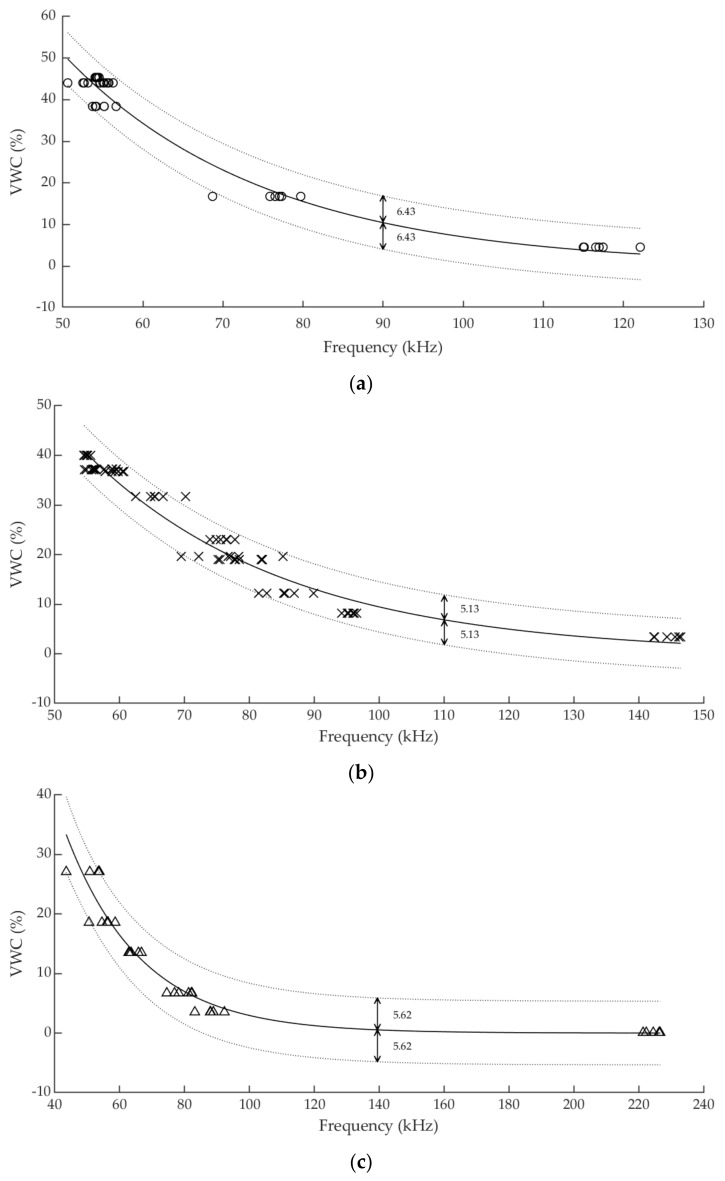
Prediction bounds for calibration fitting in: (**a**) soil (i), (**b**) soil(ii) and (**c**) soil (iii).

In order to determine the sensor accuracy to provide a VWC measurement, the fitting prediction bounds were calculated for a 95% confidence level. Thus, there is a 95% probability to find a new datum within the bounds. The exponential fitting curves together with the corresponding prediction bounds are depicted in Figure 6. The accuracy of the sensor has been found to be ±6.43, ±5.13 and ±5.62 %VWC for soils (i), (ii) and (iii), respectively, and it is associated with the half distance between upper and lower bounds. Distance between prediction bounds is not constant along the sensor working range. Therefore, considering the worst-case scenario, the greatest difference has been chosen to define the accuracy values. Compared to other commercial capacitive sensors, such as Decagon’s ECH_2_O family, which generally offers an accuracy of ±3%VWC with generic calibration and ±1–2% with specific calibration, the accuracy obtained by the experimental sensor is lower, but compensated by economic savings.

### 3.3. Sensor Experimental Validation

**Figure 7:**
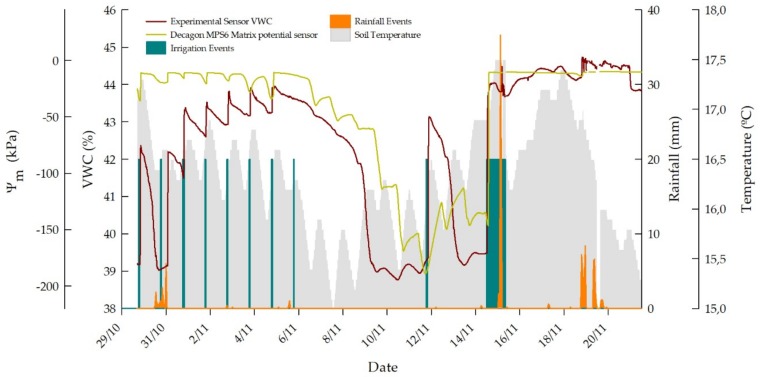
Experimental sensor and commercial MPS-6 sensor response in a sweet cherry orchard from irrigation and rainfall events.

After evaluating the response of the experimental sensor in laboratory tests, using low-volume bulk soil samples, it was tested under field conditions to assess its behavior in a real working scenario. For farmers it is not so important to know the exact value of soil moisture, but to know the evolution of water in the soil. The time it takes for water to reach a certain depth or the period during which it is retained and available for use by the crop in evapotranspiration is valuable information from the point of view of Precision Agriculture. In Figure 7 a MPS-6 and the experimental sensor evolution in a sweet cherry orchard during one month are presented. Soil temperature at 25 cm depth, measured by the MPS6 sensor, is also plotted. The experimental sensor was configured to use the soil (ii) calibration curve for calculating VWC from frequency. Irrigation and rainfall events were experimented along the test period.

During the first test week, irrigation was scheduled every day at 6 p.m. Irrigation events took 75 min with except to the third and the seventh days, when the irrigation ran for two hours. The water supply is then suspended until almost a week later, when a new event is assessed. On November 14th a too prolonged irrigation event occurred as a result of a problem with the water supply system. 

Experimental sensor measurements ranged between 39 and 45% VWC along the test period. The soil is expected to have the saturation level at 40%, as determined in laboratory tests for soil (ii). However, two variation causes can be drawn. On the one hand, the sensor accuracy for this soil has been determined to be ±5.13% VWC, so that the readings performed are in range. On the other hand, there is a greater likelihood for the soil compaction to be greater than that achieved in laboratory tests, then extending the saturation level. Moreover, substantial differences between the structures of the soil sample used in the laboratory and the soil from the field tests are evident, due to the heterogeneity of the soil.

Generally, the experimental sensor detects the irrigation events perfectly, between one and two hours after the irrigation is started; one and a half hour, typically. Rainfall events are also well detected, even when the soil is saturated, as on November 15th. The second irrigation event occurs simultaneously with several consecutive rainfalls, so that the irrigation detection is disguised in a certain way, but water infiltration is well detected anyway, either from irrigation or rain. The sensor response trend changes just after the second irrigation and a sharp increase is achieved after a pronounced rain peak. In this case, the sensor response is not as quick as in all other cases. However, a similar behavior is presented by the MPS-6 sensor. This one does not provide the VWC measurement, but the soil matrix potential. Nevertheless, water detection is equally well performed by both variables. In the case of the eighth irrigation event, i.e. on November 5th, irrigation is not detected by the experimental sensor and neither by the MPS-6. This phenomenon is likely due to the fact that the measurement influence volume of the sensors was not reached by water.

Regarding the evolution of soil temperature, a cyclical behavior is shown. Soil temperature normally starts to rise from midday onwards and the maximum is reached before midnight. Irrigation events are usually matched to temperature increases, although it is only a coincidence as a result of soil thermal inertia. Nonetheless, at no rain nor irrigation periods, such as November 6th–11th and November 13th–14th, either the experimental sensor and the MPS-6 one experiment fluctuations on their VWC and potential measurements, respectively. Fluctuations are certainly more pronounced in the MPS-6 during the former cited period, and matches perfectly the changes in soil temperature. Therefore, a soil temperature influence on both sensors response is noticed, as reported in [75,76,77] for capacitive soil moisture sensors.

## 4. Conclusions

The design, development and calibration of a new intelligent low-cost soil moisture sensor is presented in this paper. The sensor is able to operate either with analog or digital SDI-12 outputs. The two main components of the sensor (oscillator and microcontroller) have been chosen because they are general-purpose items, which guarantees their high temporary availability for manufacturing. In addition, their market price is low, which allows us to assume a low cost of the final product. It has been proved that capacitive sensors can be effectively used for soil moisture measurement and a cost reduction is feasible while keeping enough accuracy. Measurement alterations were noticed when other sensors were nearly installed, so that capacitive sensors where gathered to suffer parasitic capacities and earth leakage currents if measurement terminals were not isolated. The calibration procedure confirmed the influence of the soil texture in dielectric sensors response. Therefore, the possibility of selecting the calibration curve or adding new ones without removing the sensor from the soil, provides the sensor greater flexibility and ease of use when being adapted to a new soil. Other commercial sensors provide this possibility, but at a much higher cost. The exponential calibration curve was find to be the most suitable to explain the experimental sensor behavior in soil, giving the sensor a higher sensibility as VWC is increased. The sensor can effectively work between dry and saturation states of the soil, losing operability over saturation. The sensor accuracy has been determined to be lower than similar commercial sensors. However, the cost reduction is considerable and admissible, taking into account that localized irrigation and rainfall detection at a certain depth and soil moisture storage time provide more useful information than the very own VWC value. The field experimental tests have demonstrated that the experimental sensor can efficiently detect irrigation and rainfall events, presenting a similar behavior to that a commercial sensor, and that capacitive sensors response is slightly affected by soil temperature. Consequently, in future works the sensor presented in this paper will be fitted with a temperature detector to perform a temperature correction.

## Figures and Tables

**Table 1:** ANOVA test results and variability estimators.

Soil	VWC Level	*p*-Value	σ_sensor _(kHz)	%Repeatability	%Reproducibility
i	1	0.39236	2.65818	97.33	2.67
	2	0.22629	3.95258	74.24	5.76
	3	0.95760	1.39010	75.06	24.94
	4	0.14010	1.78168	79.28	20.72
	5	0.83585	0.20544	80.76	19.24
ii	1	0.85086	2.36019	75.76	24.24
	2	0.21983	0.89163	72.96	27.04
	3	0.60834	3.62905	81.26	18.74
	4	0.69355	3.30620	82.83	17.17
	5	0.73319	6.76834	77.59	22.41
	6	0.39447	1.38040	97.04	2.96
	7	0.12632	2.84352	52.53	47.47
	8	0.65849	1.33800	79.52	20.48
	9	0.63688	1.81779	80.22	19.78
	10	0.33364	0.76075	93.55	6.45
	11	0.81634	0.45644	76.17	23.83
iii	1	0.24829	2.40258	78.47	21.53
	2	0.77282	3.70001	76.83	23.17
	3	0.96216	4.08861	75.05	24.95
	4	0.99348	2.16998	75.00	25.00
	5	0.14704	2.97047	57.44	42.56
	6	0.24207	4.23144	77.29	22.71

**Table 2:** Parameters of calibration functions, determination coefficient (R^2^) and root mean square error (RMSE).

Soil	Function	a	b	c	R^2^	RMSE
i	$\mathrm{Exponential}\;(VWC\left(\%\right)=a\cdot e^{b\cdot f}$)	368.9	−0.03958	-	0.9653	2.9903
	Rational (VWC(%)=(a·f+b)/(f+c))	−15.26	2149	−23.52	0.9618	3.1843
	$\mathrm{Potential}\;(VWC\left(\%\right)=a\cdot f^{b}$)	1.746E6	−2.657	-	0.9614	3.1545
ii	Exponential	236.5	−0.0322	-	0.9593	2.5034
	Rational	−15.42	2391	−16.71	0.9516	2.7509
	Potential	3.077E5	−2.227	-	0.9460	2.8838
iii	Exponential	215.6	−0.04287	-	0.9237	2.6317
	Rational	−5.957	941.7	−23.52	0.8831	3.3075
	Potential	4.567E5	−2.509	-	0.8959	3.0749

f, frequency (kHz).

**Scheme A1:**
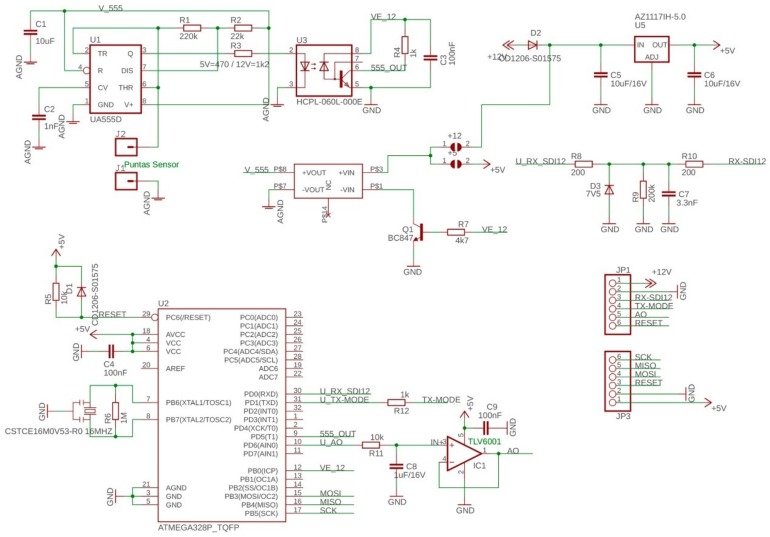
Experimental sensor schematic.
